# Efficacy and safety of remimazolam tosilate in elderly patients undergoing general anesthesia for bronchoscopy. A randomized controlled trial

**DOI:** 10.1186/s12871-025-03318-1

**Published:** 2025-11-04

**Authors:** Dongsheng Hua, Shuqing Jin, Keting Min, Jiong Song, Shihua Pei, Yuping Li, Ruowang Duan

**Affiliations:** 1 Department of Anesthesiology, Shanghai Pulmonary Hospital, School of Medicine, Tongji University, 507 Zhengmin Road, Shanghai, 200433 China; 2 Department of Anesthesiology, The First People’s Hospital of Xundian Hui and Yi Autonomous County, Yunnan, China; 3 Department of Thoracic Surgery, Shanghai Pulmonary Hospital, School of Medicine, Tongji University, 507 Zhengmin Road, Shanghai, 200433 China

**Keywords:** Remimazolam, Bronchoscopy, Elderly patients, General anesthesia

## Abstract

**Background:**

Remimazolam, a novel ultrashort-acting benzodiazepine, has been successfully used for the induction and maintenance of procedural sedation and general anesthesia. It may provide effective general anesthesia for elderly patients undergoing bronchoscopy.

**Objective:**

To evaluate the safety and efficacy of remimazolam tosilate versus propofol in elderly patients undergoing general anesthesia for bronchoscopy.

**Design:**

Prospective, single-blind, randomized clinical trial.

**Setting:**

Single-center study conducted from November 2023 to April 2024.

**Patients:**

118 elderly patients (≥ 65 years) undergoing general anesthesia for bronchoscopy.

**Interventions:**

Patients were randomly assigned 1:1 to receive propofol or remimazolam.

**Main outcome measures:**

The primary outcome was anesthesia success rate, defined as successful bronchoscopy completion with a Modified Observer’s Assessment of Alertness/Sedation (MOAA/S) score of 0 without rescue anesthesia. Secondary outcomes included vital signs, anesthesia characteristics, and adverse events.

**Results:**

There was no significant difference in the anesthesia success rate between the two groups (*P* = 0.559). The onset time, MOAA/S score at extubation, hypertension, hypoxemia, tachycardia, and bradycardia were similar between the groups (*P* > 0.05), as were emergence agitation, nausea/vomiting, and intraoperative awareness (*P* > 0.05). Compared to propofol, remimazolam was associated with significantly shorter durations of hypotension, reduced injection pain, and faster awakening time (*P* < 0.01).

**Conclusion:**

Remimazolam tosilate demonstrated non-inferior safety and efficacy to propofol for general anesthesia during bronchoscopy in elderly patients. Remimazolam was associated with a lower incidence of injection pain and hypotension. Additionally, when reversed with flumazenil, remimazolam provided significantly faster recovery times compared to propofol, potentially enhancing bronchoscopy efficiency.

**Clinical trial number:**

Clinical trial number and registry URL: ChiCTR2300076845, registration date: October 22, 2023; http://www.chictr.org.cn.

## Introduction

Bronchoscopy plays an important role in the diagnosis and treatment of lung diseases [1]. General anesthesia is now widely used in bronchoscopy, which can effectively alleviate patients’ anxiety and discomfort [2]. Propofol, known for its rapid onset of action, is the most commonly used intravenous anesthetic for bronchoscopy in clinical practice. However, propofol still has several limitations, including injection pain, hypotension, respiratory depression, a narrow therapeutic index, and the lack of specific antagonists [3]. Elderly patients demonstrate increased drug sensitivity due to the decline in physiological functions. Nevertheless, the monitoring and medication management during bronchoscopy in elderly patients are similar to those in adults, which enhances the risk of perioperative adverse events [4]. Therefore, the development of a new intravenous anesthetic that can compensate for the above shortcomings remains an important clinical need.

Remimazolam is one of the novel ultra-short-acting anesthetics [5]. Remimazolam can enhance the activity of GABAA receptors with γ-subunits, leading to chloride ion influx and inducing neuronal membrane hyperpolarizationto to inhibit neuronal activity [6]. Remimazolam exhibits a rapid onset of action (1–3 min) and a short elimination half-life (approximately 0.75 h). Flumazenil can effectively reverse the effects of remimazolam [7]. A prior study compared the safety of remimazolam and propofol in adult patients undergoing moderate sedation during bronchoscopy [8]. Nevertheless, the patient age distribution in that study was broad, and there is currently no research specifically investigating the effects of remimazolam in elderly patients undergoing general anesthesia for bronchoscopy. The purpose of this study is to evaluate the safety and efficacy of remimazolam tosilate in elderly patients undergoing general anesthesia during bronchoscopy.

## Materials and methods

### Study design

This was a single-center, prospective, single-blind, randomised clinical trial. The trial took place at the Department of Endoscopy, Shanghai Pulmonary Hospital, affiliated with School of Medicine, Tongji University, from November 2023 to April 2024. Participants were briefed on the study’s objectives, and written informed consent was acquired prior to the procedure. The trial received approval from the Ethics Committee of Shanghai Pulmonary Hospital (identifier: L23-001-1) and was prospectively registered at www.chictr.org.cn. (ChiCTR2300076845).

### Patients

Patients scheduled to undergo bronchoscopy with general anesthesia were randomly assigned to the propofol group (Group P) or the remimazolam group (Group R) in a 1:1 ratio.

Inclusion criteria: age ≥ 65 years; ASA: I-III; SpO2 > 90% (breathing air); Ability to understand and voluntarily sign the informed consent form and willing to complete the study according to the requirements of the study protocol.

Exclusion criteria: severe liver, kidney, or respiratory dysfunction; allergy to remimazolam, propofol, alfentanil, flumazenil or rocuronium; current mechanical ventilation; current treatment with opioid or benzodiazepine medications; substance abuse (drug or alcohol); cognitive impairment.

### Protocol

The preoperative fasting was routinely required for 6–8 h for solids and 4 h for liquids. Thirty minutes before the procedure, the patient’s basic information was collected, including gender, age, height, weight, ASA classification, current medical history and treatment, past medical history, allergy history, and baseline vital signs (heart rate, blood pressure, and oxygen saturation).

Upon entering the procedure room, intravenous access was established, followed by 2 min of mask oxygenation at a flow rate of 6 L/min. An intravenous injection of 10 µg/kg alfentanil was administered, and 3 min later, patients in the R group received 0.1 mg/kg of remimazolam intravenously, while those in the P group received 2.0 mg/kg of propofol for anesthetic induction, with the injection completed within 1 min. Loss of consciousness (LoC) was defined as a MOAA/S score of ≤ 1, with no response to shoulder shaking following the initiation of the study drug administration. After confirming LoC, 0.6 mg/kg of rocuronium bromide was administered intravenously, followed by tracheal intubation and assisted ventilation. Respiratory parameters were adjusted to achieve a tidal volume of 6–10 ml/kg and a respiratory rate of 10–16 breaths per minute, maintaining end-tidal carbon dioxide (EtCO2) levels between 35 and 45 mmHg.

In the R group, remimazolam was continuously infused at a rate of 1.0-2.0 mg/kg/h, while in the P group, propofol was infused at 3.0-4.0 mg/kg/h to maintain anesthesia. The infusion rates were adjusted within the prescribed maintenance dose range, guided by the intensity of surgical stimuli. If signs such as movement, sweating, lacrimation, eye opening, coughing, or other symptoms of inadequate anesthesia occurred, patients in the R group received an additional intravenous dose of 0.1 mg/kg remimazolam, or patients in the P group received 1.0 mg/kg propofol as a rescue anesthetic. If hemodynamic instability persisted, the anesthesiologist intervened with appropriate vasoactive drugs such as nicardipine or esmolol based on their clinical judgment.

At the end of the surgery, all patients received an intravenous injection of 2 mg/kg sugammadex to reverse muscle relaxation and cease all medications. Patients in the remimazolam group also received an additional intravenous dose of 0.5 mg of flumazenil. The patients were considered awake when spontaneous breathing returned and could follow instructions to complete movements, followed by tracheal extubation. The patients were then transferred to the recovery room. Hemodynamic data (heart rate, blood pressure) were collected at four time points: before anesthesia induction (T1), 1 min after anesthesia induction (T2), 10 min after anesthesia induction (T3), and upon arrival in the PACU (T4).

### Efficacy assessment

The primary outcome was the anesthesia success rate, defined as the completion of bronchoscopy while maintaining a MOAA/S score of 0 and without the need for rescue anesthesia.

Secondary outcomes included safety indicators and clinically relevant parameters. Safety indicators encompassed: bradycardia (heart rate < 50 beats/min or a decrease of more than 20% from baseline for at least 30 s); tachycardia (heart rate > 120 beats/min or an increase of more than 20% from baseline for at least 30 s); hypotension (systolic blood pressure < 80 mmHg, diastolic blood pressure < 40 mmHg, or a decrease of more than 20% from baseline); hypertension (systolic blood pressure > 180 mmHg, diastolic blood pressure > 100 mmHg, or an increase of more than 20% from baseline); hypoxemia (oxygen saturation < 90% for more than 1 min). Additionally, clinically relevant parameters included onset time (from drug administration to achieving MOAA/S = 0); recovery time (from the completion of bronchoscopy to extubation); MOAA/S score at the time of extubation; incidence of delayed recovery and agitation during the recovery period; incidence of intraoperative awareness and other adverse events; and the occurrence of injection pain.

### Sample size estimation

The study employed a parallel-controlled design, with the sample size calculation based on the results of two significant clinical trials: a multicenter, randomized, double-blind, parallel-controlled trial published in Chest journal in 2019 [9], and a multicenter, randomized, double-blind, positive-controlled trial published in Front Pharmacol journal in 2022 [8]. The sedation success rate for remimazolam during general anesthesia for fiberoptic bronchoscopy was 80.6%, compared to 99.4% for the propofol group. A two-sided test was applied with a significance level (α) of 0.05 and a power (1-β) of 0.9. Considering a 20% dropout rate, the required sample size was calculated to be 118 cases using PASS 21.0 software, with 59 participants in the remimazolam group and 59 in the propofol group.

### Statistical analysis

The analysis was performed using SPSS 26.0 statistical software. Continuous data were expressed as mean ± standard deviation (Mean ± SD) or median (interquartile range, IQR). Categorical data were presented as counts (percentages). The χ² test or Fisher’s exact test was used to compare categorical variables. For continuous variables, repeated-measures ANOVA was used for multiple-group comparisons, and independent sample t tests were used for between-group comparisons. Statistical significance was set at *P* < 0.05.

## Results

### Recruitment and baseline characteristics of subjects

**Fig. 1:**
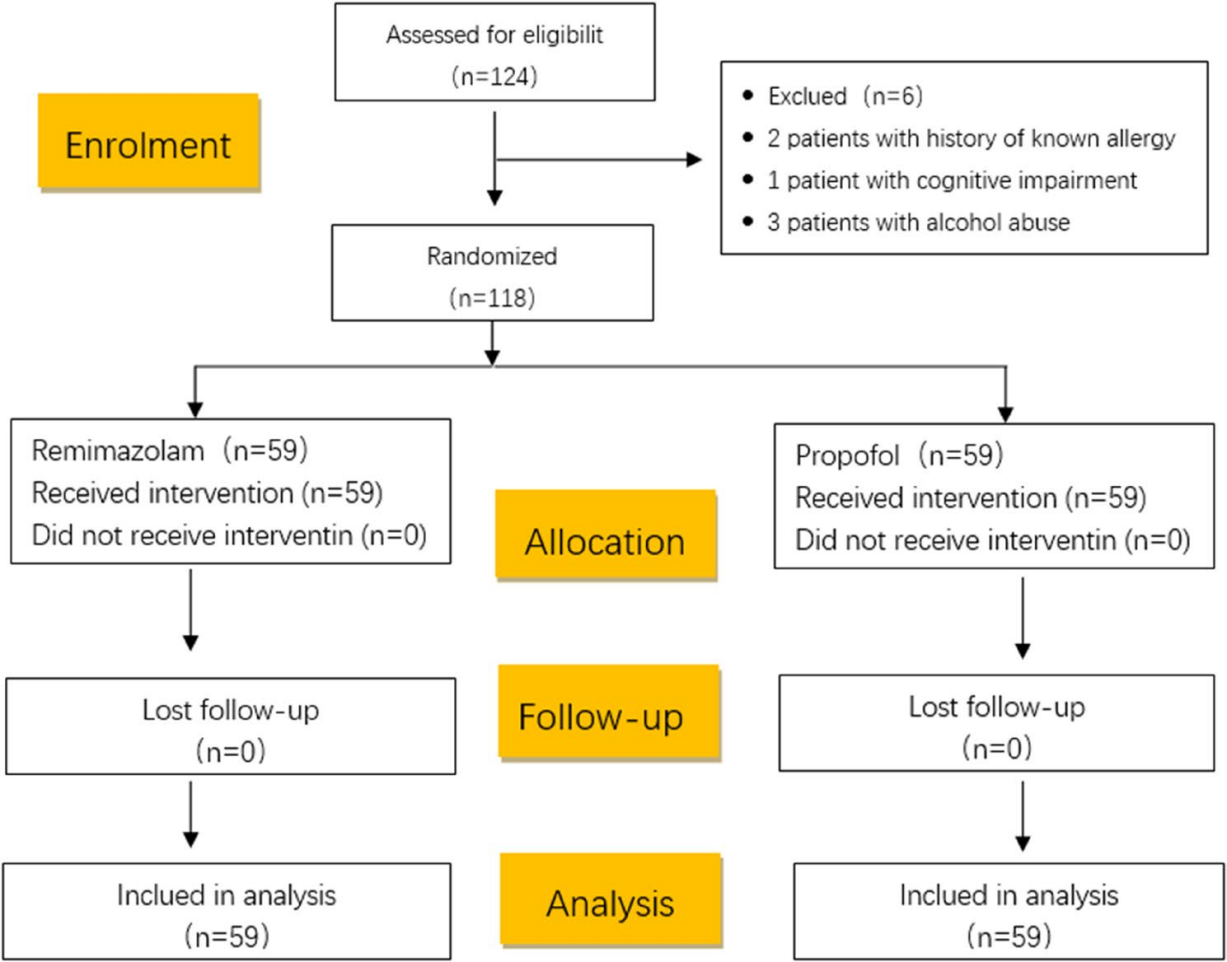
CONSORT diagram

A total of 124 elderly patients were recruited for the study from November 2023 to January 2024. Among them, 6 participants were excluded from the trial for the following reasons: 2 patients had a known history of allergies; 1 patient had cognitive impairment; and 3 patients had a history of alcohol addiction. Consequently, 118 participants were included and assigned to two groups, completing the trial (Fig. 1). There were no significant differences in demographic characteristics and baseline characteristics between the two groups (*P* > 0.05) (Tables 1 and 2).

**Table 1:** Demographic data

	Remimazolam (*n* = 59)	Propofol (*n* = 59)	*P* Value
Gender			
Male	37(62.7%)	44(74.6%)	0.165
Age (years)	70.00(5.00)	71.00(6.00)	0.707
Height (cm)	163.64 ± 9.92	164.00 ± 7.50	0.826
Weight (kg)	61.32 ± 13.93	62.98 ± 9.79	0.455
BMI (kg/m^2^ )	22.76 ± 4.12	23.43 ± 3.34	0.339
SBP (mmHg)	156.15 ± 20.92	158.22 ± 26.01	0.635
DBP (mmHg)	81.15 ± 10.88	84.31 ± 12.23	0.142
MAP (mmHg)	109.81 ± 13.42	110.92 ± 16.35	0.690
HR (bpm)	81.32 ± 15.70	81.29 ± 15.15	0.991
SPO_2_(%)	96.00 ± 1.965	96.51 ± 2.480	0.220

Data are presented as the means (95% confdence intervals), medians (interquartile ranges, or number (percentage) of patients; *BMI *Body mass index, *SBP *Systolic blood pressure, *DBP *Diastolic blood pressure, *MAP *Mean arterial pressure, *ASA *American Society of Anesthesiologists, *HR *Heart rate, *SpO2 *Oxygen saturation

**Table 2:** Analysis of bronchoscopy type and duration

	Remimazolam (*n* = 59)	Propofol (*n* = 59)	*P* Value
Types of bronchoscopy			
EBUS (*n*,%)	38(64.40%)	50(84.75%)	
Biopsy (*n*,%)	16 (27.12%)	8 (13.56%)	
Intraluminal treatment (*n*,%)	5 (8.47%)	1 (1.69%)	
Duration of bronchoscopy (min)	20.29 ± 7.57	18.51 ± 7.13	0.191

Data are presented as the means (95% confdence intervals), medians (interquartile ranges, or number (percentage) of patients; EBUS: Endobroncheal Ultrasonography

### Efficacy assessment

#### Primary outcome

The anesthesia success rate was 96.6% in Group R and 98.3% in Group P, demonstrating no statistically significant difference between the two groups with regard to the primary outcome. (*P* = 0.559) (Table 3).

**Table 3:** Proportional analysis of sedation success

	Remimazolam (*n* = 59)	Propofol (*n* = 59)	*P* Value
Sedation success rate (*n*,%)	57(96.61%)	58(98.31%)	0.559
Induction period (*n*,%)	57(96.61%)	58(98.31%)	0.559
Maintenance period (*n*,%)	59(100%)	59(100%)	>0.999

Data are presented as the means (95% confdence intervals), medians (interquartile ranges , or number (percentage) of patients

#### Sencondary outcomes

##### Safety indicators

**Fig. 2:**
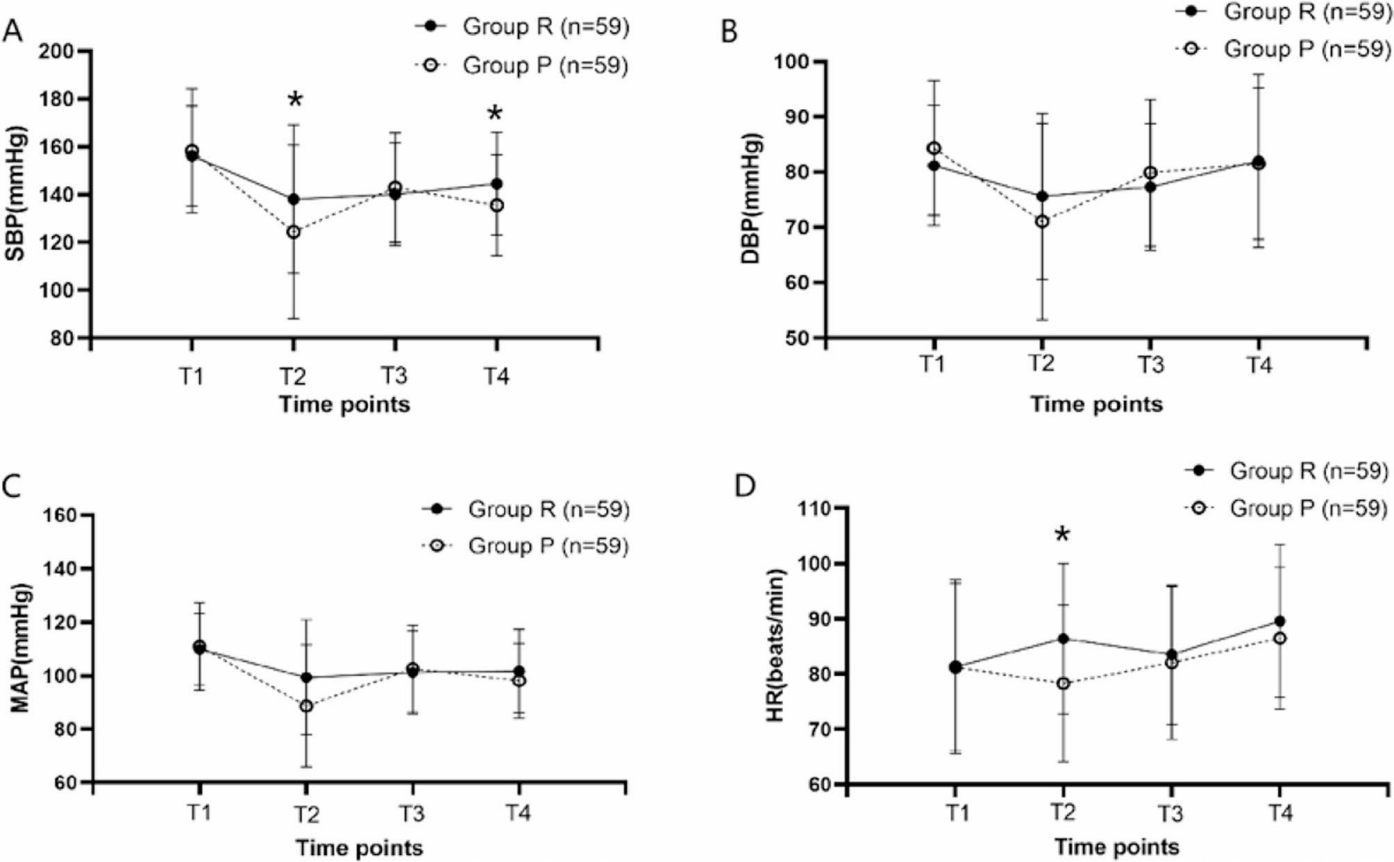
Hemodynamics. **A** Systolic Blood Pressure (SBP), **B** Diastolic Blood Pressure (DBP), **C** Mean Arterial Pressure (MAP), **D** Heart Rate (HR). Group P: group receiving propofol, Group R: group receiving remimazolam. T1, before anesthesia induction; T2, 1 min after anesthesia induction; T3, 10 min after anesthesia induction.T4, entry into the PACU

In the analysis of safety indicators, there were no significant differences in the incidence of hypertension, tachycardia, bradycardia, or hypoxemia between the two groups and the HR in group R was significantly higher than that in group P at T2 (*P*<0.05). However, the incidence of hypotension was significantly lower in Group R compared to Group P(*P* = 0.001). A total of 30 patients in Group R and 45 patients in Group P experienced hypotension. A significant difference in the incidence of hypotension was observed between the two groups during both the induction period (*P* = 0.000) and the recovery period (*P* = 0.002) (Table 4) (Fig. 2).

**Table 4:** Safety indicators

	Remimazolam (*n* = 59)	Propofol (*n* = 59)	*P* Value
Hypertension (*n* ,%)	32(54.24%)	34(57.63%)	0.711
Induction period (*n*,%)	10(16.95%)	9(15.25%)	0.802
Maintenance period (*n*,%)	20(33.90%)	24(40.68%)	0.446
Recovery period (*n*,%)	8(13.56%)	13(22.03%)	0.229
Hypotension (*n*,%)	30(50.85%)	45(76.27%)	0.004
Induction period (*n*,%)	19(32.20%)	37(62.71%)	0.001
Maintenance period (*n*,%)	17(28.81%)	20 (33.90%)	0.552
Recovery period (*n*,%)	9 (15.25%)	21 (35.59%)	0.011
Tachycardia (*n*,%)	28 (47.46%)	22 (37.29%)	0.264
Induction period (*n*,%)	15 (25.42%)	7 (11.86%)	0.059
Maintenance period (*n*,%)	9 (15.25%)	11 (18.64%)	0.624
Recovery period (*n*,%)	17 (28.81%)	14 (23.73%)	0.530
Bradycardia (*n*,%)	4 (6.78%)	10 (16.95%)	0.088
Induction period (*n*,%)	1 (1.69%)	6 (10.17%)	0.119
Maintenance period (*n*,%)	0	5 (8.47%)	0.068
Recovery period (*n*,%)	3 (5.08%)	1 (1.69%)	0.611
Hypoxemia (*n*,%)	1(1.69%)	0	1.000

Data are presented as the means (95% confidence intervals), medians (interquartile ranges , or number (percentage) of patients

##### Clinical indicators

**Fig. 3:**
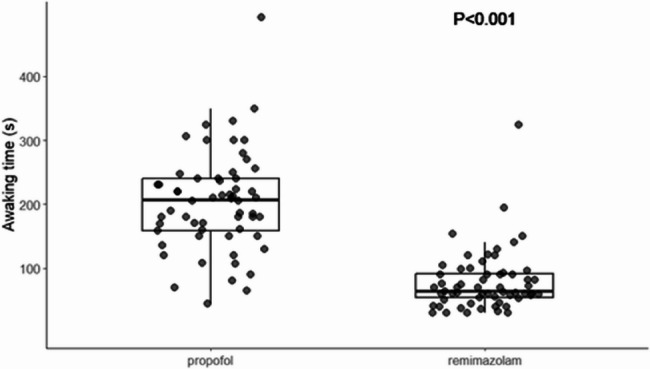
Awaking time from general anaesthesia

There were no significant differences between the two groups in terms of the MOAA/S score at extubation, onset time, intraoperative awareness, nausea and vomiting, or agitation during the recovery period (*P* > 0.05). However, the incidence of injection pain was significantly lower in Group R compared to Group P (16.95% vs. 45.76%, *P* = 0.001). Furthermore, when remimazolam was administered intravenously in combination with flumazenil, the awakening time was significantly shorter than that in the propofol group (*P* < 0.01) (Table 5) (Fig. 3).

**Table 5:** Clinical indicators

	Remimazolam (*n* = 59)	Propofol (*n* = 59)	*P* Value
Onset time(s)	33.00(10.00)	40.00(20.00)	0.129
Awaking time(s)	64.00(40.00)	206.00(82.00)	0.000
Extubation MOAA/S score	5.00(0)	5.00(0)	0.225
Injection pain (n,%)	10(16.95%)	27(45.76%)	0.001
Intraoperative awareness (n,%)	0	0	1.000
Nausea and vomiting (n,%)	0	0	1.000
Awaking agitation (n,%)	0	0	1.000

Data are presented as the means (95% confidence intervals), medians (interquartile ranges , or number (percentage) of patients

## Discussion

This trial demonstrates that remimazolam tosilate provides non-inferior anesthesia efficacy compared to propofol for bronchoscopy in elderly patients, with distinct advantages in hemodynamic stability and recovery profile. Notably, remimazolam significantly reduced the incidence of hypotension and injection pain, while its reversal with flumazenil accelerated awakening by more than 2 min. Therefore, remimazolam tosilate presents a promising anesthesia option for elderly patients undergoing bronchoscopic procedures.

Our anesthesia success rates align with studies involving elderly patients undergoing hysteroscopy [10]. The shorter loss-of-consciousness (LoC) time with remimazolam contrasts with prior data on adults [11, 12]. The recommended induction dose of remimazolam for adult Chinese patients undergoing general anesthesia is 0.2 mg/kg/min [13], and there is currently no recommended dosage for elderly patients. Doi et al.‘s study indicated that induction doses of 6 mg/kg/h and 12 mg/kg/h for remimazolam are not inferior to 2.0-2.5 mg/kg/min of propofol [14]. When remimazolam is used at the same dose for induction, elderly patients experience a shorter LoC than younger patients [15]. The effective dose for 95% of patients (ED95) required to achieve LoC decreases with age [16]. Since the participants in our study were elderly, this may explain the shorter LoC observed with remimazolam.

Injection pain is a common adverse reaction associated with propofol, with an incidence rate reaching up to 66% [17], primarily due to venous endothelial irritation caused by its aqueous phase [18]. To mitigate this issue, clinicians commonly administer pretreatments such as lidocaine, nonsteroidal anti-inflammatory drugs, and opioids. In contrast, remimazolam tosilate’s non-phenolic structure reduces tissue irritation [19], which aligns with our findings of a lower risk of injection pain compared to propofol [12, 20].

Consistent with prior studies, hypotension was prevalent during bronchoscopy under anesthesia [21]. Remimazolam demonstrated a lower incidence of hypotension than propofol during both induction and maintenance, while hypertension and bradycardia showed no significant differences [22, 23]. Propofol-induced hypotension involves decreased myocardial contractility, reduced venous return, and systemic arterial resistance [24–26]. Previous research has also indicated that propofol results in a lower incidence of tachycardia compared to benzodiazepines [27], potentially due to myocardial inhibition counteracting bronchoscopy-induced catecholamine release [28, 29]. Although our study noted tachycardia disparities during the induction period, these were not statistically significant. Remimazolam’s safety profile extends to high-risk and pediatric populations [30], with potential anxiolytic benefits [31].

Respiratory depression remains a critical concern in spontaneously breathing bronchoscopy [32], where shared airway challenges exist. Studies have confirmed that remimazolam causes less respiratory depression and hypoxemia than propofol in such settings [33, 34], though these advantages were not evident in our mechanically ventilated cohort.

While remimazolam alone may not shorten awakening time [22, 35], flumazenil reversal significantly accelerates recovery [36, 37], enhancing procedural efficiency. However, routine use of the antagonist warrants caution due to the potential for rebound anxiety [37].

Our study has several limitations:1. Operator blinding was not feasible due to the distinctive appearance of propofol. 2. Non-invasive cuff monitoring of patients’ blood pressure may lead to delayed reflection of blood pressure changes, potentially impacting the accuracy of the measurements. 3. Baseline blood pressure readings for patients undergoing fiberoptic bronchoscopy in outpatient settings were challenging to obtain accurately. Consequently, baseline blood pressure measurements relied on readings taken before the procedure from ostensibly calm patients. Notably, preoperative nervousness or anxiety in some patients might have led to a elevated baseline blood pressure readings. 4.This study is a single-center study, and its results may have limited generalizability. Further validation in other centers is required. 5. This study did not utilize objective monitoring of anesthetic depth, such as BIS, which may have influenced the trial results.

## Conclusion

The use of remimazolam tosilate for anesthesia in elderly patients undergoing bronchoscopy with general anesthesia demonstrates both safety and effectiveness. The incidence of injection pain and hypotension is lower compared to propofol, and the combination of remimazolam tosilate with flumazenil significantly reduces the awakening time compared to propofol.

## Data Availability

The datasets generated and analyzed during the current study are available from the corresponding authors on reasonable request.

## Abbreviations

MOAA/S: Modified Observer’s Assessment of Alertness/Sedation Score
LoC: Loss of Consciousness
ASA: American Society of Anesthesiologists
EtCO2: End-tidal Carbon Dioxide
PACU: Postanesthesia Care Unit
MBP: Mean Blood Pressure
SBP: Systolic Blood Pressure
DBP: Diastolic Blood Pressure
HR: Heart Rate
